# Effectiveness of physical exercise in the treatment of depression in older adults as an alternative to antidepressant drugs in primary care

**DOI:** 10.1186/s12888-018-1982-6

**Published:** 2019-01-14

**Authors:** Jesús López-Torres Hidalgo, the DEP-EXERCISE Group, Luis Aguilar Salmerón, Clotilde Boix Gras, Monchi Campos Rosa, Francisco Escobar Rabadán, Concepción Escolano Vizcaíno, José Luis Estellés Belenguer, Juan Fernández Martín, Vicente Ferrer López, Almudena Legido García, Jaime López-Torres López, Jesús López-Torres López, Maria Ángeles López Verdejo, Ana López Yeste, María Ángeles Lloret Callejo, María Jesús Montes Lozano, Juana Muñoz Núñez, Karen Nieto Rodríguez, Joseba Rabanales Sotos, Isabel Rodenas García, Carmen Somoza Castillo, Juan Manuel Téllez Lapeira

**Affiliations:** Albacete Zone VIII Health Centre and Faculty of Medicine, Madrid, Spain

**Keywords:** Depression, Elderly, Exercise

## Abstract

**Background:**

Although currently available evidence suggests that physical exercise can be beneficial for depressed patients and might be comparable to antidepressant treatment, the best way of implementing this recommendation in clinical practice is not known. This study therefore aims to ascertain the non-inferiority of supervised physical exercise to antidepressant drug treatment, in terms of reducing depressive symptoms among patients presenting with clinical criteria of a depressive episode (ICD-10), across a follow-up period of 6 months.

**Methods:**

It will take the form of a randomised clinical trial undertaken in a primary care setting, in which a total of 312 patients over the age of 65 years with clinically significant depression will be randomly assigned to supervised physical exercise programme, or will alternatively receive treatment with antidepressant drugs habitually used in clinical practice. Participants’ physical condition will be assessed at baseline, and again at 15 days and 1, 3 and 6 months. The supervised exercise programme will consist of 2 weekly sessions in groups of 10–12 patients across a period of 6 months, in which a sports instructor will train patients to do at least 30 min of regular activity at moderate intensity on an almost daily basis, including aerobic, muscle-strengthening, flexibility, and balance-strengthening exercises. The following will be assessed at regular intervals in both groups: status of depression symptoms; level of physical activity; self-perceived health status; appearance of adverse effects; and adherence to the physical exercise programme or antidepressant treatment. The principal outcome variable will be a reduction in pre-treatment depression-symptom scale scores (Montgomery-Asberg Depression Rating Scale and Geriatric Depression Scale).

**Discussion:**

In terms of the number of patients and duration of follow-up, this proposed clinical trial is a project which easily surpasses the few studies on this subject that have been previously conducted on the elderly. Its aim is to provide solid scientific evidence on a therapeutic resource -physical exercise- which has undeniable health benefits and can be applied to certain health problems, such as depressive disorders, which are of great magnitude and considerable socio-economic relevance, and have a significant impact on the quality of life of older adults.

**Trial registration:**

ClinicalTrials.gov NCT03358433 (retrospectively registered on 11/25/2017).

## Background

Currently, there is overwhelming evidence to show the health benefits of physical exercise [1]. The Global Burden of Disease study has classified sedentarism as the fifth leading cause of disease burden in Western Europe and as one of the main modifiable risk factors [2]. Yet, despite recommendations to promote exercise as a strategy capable of reducing the burden of chronic diseases, the frequency and intensity of physical activity in the population are rather disconcerting. Physical activity is currently ranked among the health determinants which exert most influence on morbidity and mortality. Moreover, exercise can partially reverse the effects of ageing in physiological functions and conserve functional reserve among older adults [3].

Depression is a common and disabling condition that affects over 120 million people worldwide [4] -at least one in five people during their lifetime- and has a significant impact on health status. While it is usually treated with antidepressants and/or psychological therapy, such treatments are not effective in all cases, and increasing attention has recently been given to some alternatives, and to aerobic exercise in particular [5].

Depressive disorders among the aged are a paradigm of geriatric care in terms of the importance of prevention, differences in pathogenesis, diagnostic and therapeutic complexity, associated high risk of failure, and severe impact on quality of life. Old age is the time of life when emotional fragility is accentuated. In addition to neurobiological changes in the brain, ageing inevitably entails an important loss over the years, not only in terms of individuals’ emotions, but also in terms of their physical condition and social status. Depression is the most common psychological disorder among people over the age of 65 years and affects approximately 15% of this age group [6].

Recent years have witnessed a dramatic rise in the prescription of antidepressant drugs, which has greatly increased health spending. Over half of the total cost of depression corresponds to direct costs, with those generated by antidepressant drugs becoming increasingly relevant. These drugs have undesirable effects, especially among the older population, with their use often being continued indefinitely and unnecessarily. It would therefore seem reasonable to test new therapeutic modalities which, a priori, would have fewer adverse effects and, most likely, a lower impact on health expenditure.

Among the reasons why exercise could improve depression is the belief that, on the one hand, it could act as a distraction from negative thoughts and that, on the other, it is possibly important to master new skills. In addition, social contact could form part of this mechanism. Physical activity can have physiological effects, such as bringing about changes in endorphin and monoamine levels, or decreases in the level of cortisol, the stress hormone, which may result in an improvement in patients’ mood [7]. Recent studies suggest that exercise stimulates the growth of new nerve cells and releases proteins, e.g., brain-derived neurotrophic factor, to improve the survival of nerve cells [8, 9]. Even so, a significant degree of uncertainty surrounds the effectiveness of exercise on depression [10, 11], mainly due to methodological considerations [12].

It should be stressed that older adults tend to be underrepresented in clinical trials in which both pharmacological and non-pharmacological measures for depressive disorders are assessed. Accordingly, and because these disorders present with special characteristics and a wide degree of clinical polymorphism among the elderly (difficulty in recognising the symptoms of depression, frequent somatic complaints, etc.), it would be of interest to carry out a clinical trial exclusively on adults aged over 65 years. This population group often presents with co-morbidities and, as a result, may be undergoing poly-pharmacy treatments. It is therefore important to demonstrate the effectiveness of non-pharmacological measures, such as physical exercise, in the case of depressive disorders.

The National Institute for Clinical Excellence (NICE) [13] conducted a systematic review on the likelihood of remission, reduction of symptoms and adherence to treatment among patients with depression who did and those who did not do exercise, and the different treatments available (pharmacological, psychotherapeutic, etc.), including a total of 25 clinical trials. Although the data which compared physical activity to antidepressant drugs indicated that there were no significant differences, the confidence intervals used by the studies were very wide, with the result that there was insufficient evidence to arrive at a conclusion. Overall, however, the studies suggested a benefit for physical activity -and more specifically for group-based physical activity- in the treatment of minor depression and mild-to-moderate major depression. In addition, physical activity has the advantage of contributing other health benefits, beyond a simple improvement in symptoms of depression [14].

The Guideline of the Institute for Clinical Systems Improvement [15] considers that physical activity could be a useful tool for ameliorating the symptoms of major depression. Another systematic review [16], in which only three studies with patients suffering from major depression were included, reported that physical exercise programmes significantly reduced the symptoms of depression, though the conclusions were limited by the heterogeneity and methodological shortcomings of the three studies evaluated.

A Cochrane review [17] analysed the relationship between physical exercise and depression. An attempt was made to answer the question of whether exercise might be more effective than drugs, psychological therapy or therapeutic abstention in reducing symptoms of depression. To this end, a total of 39 clinical trials of widely differing quality were jointly analysed. The results showed that: as compared to no treatment or a control group, exercise may have a moderately beneficial effect on symptoms of depression; and as compared to pharmacological treatment or psychological therapy, no differences were in evidence (conclusions based on very few studies). In only 4 trials [18–21], which covered a total of 298 patients, was physical exercise compared to pharmacological treatment (sertraline), and no statistically significant differences were observed, though only one of these studies specifically targeted persons over the age of 65 years [21] and included only 37 of such subjects. Furthermore, in none of these studies was follow-up longer than 16 weeks. This review was an update of a previous review [22] which suggested that exercise could reduce depressive symptoms.

New research studies are needed to further detail the type of exercise that could be beneficial for older adults with depression, analyse whether such exercise could be a therapeutic alternative to antidepressants or psychological treatment, and determine the related risks and costs. Prescribing physical exercise in the consulting room, as if it was a remedy for chronic disease, calls for knowledge about which exercise is the most suitable to the task, and the duration, frequency and intensity required.

Available evidence suggests that exercise may benefit depressed patients and be comparable to antidepressant treatment [23]. It seems reasonable, therefore, for exercise to be recommended to people with depressive symptoms and those who meet diagnostic criteria for depression [24]. As yet however, the best way of implementing this recommendation in clinical practice is not known (Sonnott, 2013). It has not been possible to provide patients with accurate information about how effective exercise can be, the relative benefits of aerobic, resistance or combined exercise, whether it is better to do such exercise individually or in a group, and the optimal duration of a session. Hence, to obtain a more accurate idea of the effect of exercise on depression among older adults, there is a need for new trials based on scientifically solid methodology, an adequate number of participants, and a sufficiently long follow-up.

The aim is to ascertain the non-inferiority of supervised physical exercise to antidepressant drug treatment in terms of reducing depressive symptoms among patients presenting with clinical criteria of a depressive episode (International Classification of Diseases -10th revision/ICD-10) diagnosed in primary care.

## Methods

### Study design and setting

**Fig. 1:**
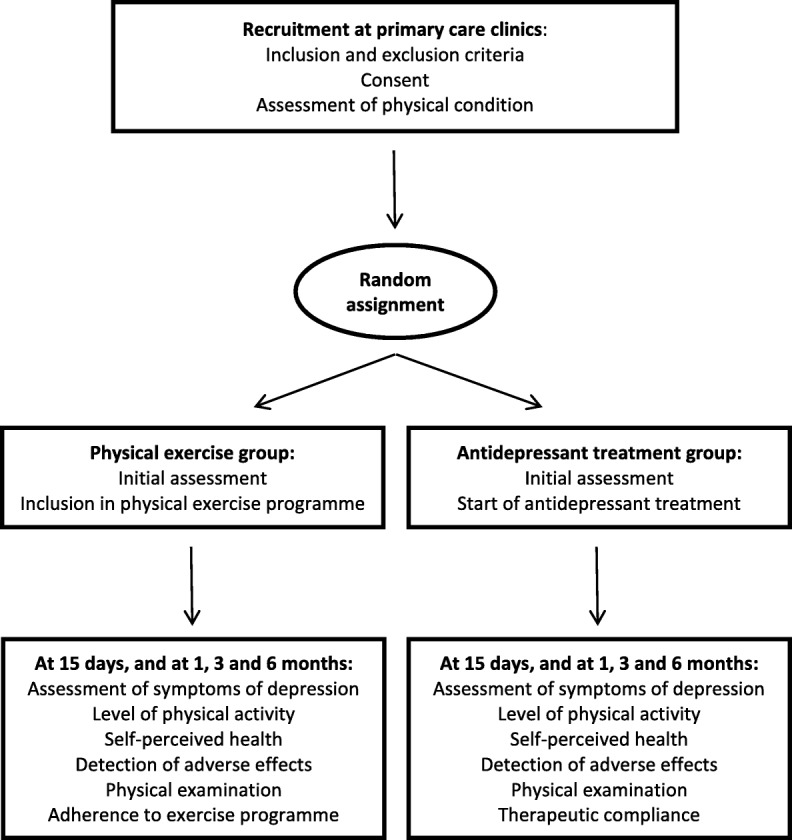
Flowchart of the study design

This will take the form of a randomised clinical trial (Fig. 1) to be conducted at health centres in the Albacete Health Area (Spain).

### Characteristics of participants

The target population will be subjects aged over 65 years with clinical criteria of a “clinically significant” depressive episode. We intend to use the ICD-10 criteria, which require a minimum of 4 out of 10 symptoms, including at least 2 of the following 3, namely, depressed mood, anhedonia and loss of energy. The 10 symptoms include: depressed mood; loss of interest or pleasure; loss of or increase in weight; insomnia or hypersomnia; agitation or slowing of movements; fatigue or loss of energy; feelings of inadequacy or guilt; poor concentration; poor self-esteem; and recurrent thoughts of death. The duration of the episode must be a minimum of two weeks.

The inclusion criteria will be the following: subjects with the above criteria of mild or moderate depressive episode belonging to the participating health centres. The exclusion criteria will be: physical or mental limitations that bar participation in the study; contraindications for doing physical training (unstable angina, arterial hypertension with systolic blood pressure > 200 or diastolic blood pressure > 110 mmHg, orthostatic hypotension > 20 mmHg accompanied by symptoms, left ventricular outflow tract due to severe/moderate aortic stenosis or obstructive hypertrophic myocardiopathy, supraventricular or ventricular arrhythmias with haemodynamic deterioration not controlled with treatment, decompensated heart failure, third-degree atrioventricular block without pacemaker implantation, and important orthopaedic problems); patients with severe depressive disorder (important interference in social or occupational functioning, psychotic symptoms, active suicidal ideation or situations of personal abandonment); depressive disorders due to a medical or substance-induced disease, depressive disorders in partial or total remission and unspecified depressive disorders; subjects with evidence of a high level of physical activity according to the International Physical Activity Questionnaire (IPAQ); and patients taking antidepressant drugs.

In all participants, initial physical condition will be measured by determining their resting heart rate with a heart-rate monitor and their submaximal heart rate, calculated from the maximum heart rate (220 - age) on an exercise bicycle (70–85% of maximum heart rate at a constant workload and speed for 6 min), electrocardiogram, and flexibility (using the sit and reach test).

### Sample size

Based on an estimated positive response in 75% of participants (a reduction of at least 50% in pre-treatment scores on the Montgomery-Asberg Depression Rating Scale/MADRS) in both the intervention (supervised physical exercise) and control groups (antidepressant therapy), an alpha risk of 0.025, a beta risk of 0.20, a non-inferiority margin of 15% (maximum difference of response for it to be deemed not inferior to the experimental treatment) and a percentage loss of 20%, a total of 156 patients will be required in each group (*n* = 312).

Participants will be consecutively selected at 20 family medicine clinics belonging to 3 health centres.

### Interventions

All participants will be observed over a period of 6 months, with assessments being made at baseline and then again at 15 days and 1, 3 and 6 months. In any case where symptoms worsen, with the patient’s clinical profile progressing from mild or moderate to severe depression, the patient will be referred to a mental health department, which will then decide on the course of treatment to be followed. The trial will be deemed to have ended in the following circumstances: completion of the observation period; patient withdrawal; or withdrawal of informed consent.

Patients will be included in the intervention group (physical exercise) or the control group (antidepressant therapy) by simple randomisation generated by a computer software programme. The randomisation sequence will be concealed throughout the recruitment period.

With respect to antidepressant drug therapy, the NICE guideline holds to the general view that there is little difference among the various antidepressants in terms of efficacy. As a result, patients’ physicians will decide on the most suitable drug in every case.

It will be suggested to subjects assigned to the intervention group that they participate in a physical exercise programme consisting of two 1-h sessions per week for a period of 6 months (a total of 48 sessions in groups of 10–12 persons), to be given by sports coaches and held at sports facilities.

The educational content of physical activity, based on the recommendations of the American College of Sports Medicine, will include the following: how to increase physical effort in activities of daily living; how to perform regular exercise adapted to one’s age and individual condition, to help maintain ideal body weight; how to do exercise or aerobic sport; how to warm up; how to perform stretching exercises (musculotendinous stretching); how to conduct a period of cooling-down exercises and relaxation; and how to approach muscle-strengthening and flexibility training.

Participants will be shown how to increase their levels of physical activity in daily life, with the idea that at least 30 min of average to moderately intense activity should be performed throughout the entire follow-up period on an almost daily basis. The physical exercise programme will include: aerobic exercises (goal: a minimum of 30 min of aerobic activity of moderate intensity five days per week); muscle-strengthening exercises (goal: a minimum of two non-consecutive days per week, with 10–15 repetitions of each exercise at a moderate-to-high level of intensity); flexibility exercises (goal: at least twice per week for at least 10 min); and balance-strengthening exercises (goal: at least three times per week).

The educational intervention will be conducted from a behavioural standpoint, aimed at achieving patients’ understanding and acceptance, and bringing about a shift towards improvement in their habits. The following will be required: a baseline for adopting and maintaining lifestyle changes; patients’ commitment to and an active role in the process; and a linear design, with consecutive phases of learning adapted to needs.

### Study variable

The principal outcome variable will be a reduction in depressive symptoms: a fall in the pre-treatment 10-item Montgomery-Asberg Depression Rating Scale (MADRS) and 15-item Geriatric Depression Scale (GDS) scores, with these scales being administered at baseline and subsequently at 15 days and 1, 3 and 6 months.

At baseline, the following variables will be assessed in all participants: health problems (as classified by the International Classification of Primary Care/ICPC-2); drug use (Anatomical Therapeutic Classification); toxic habits (consumption of tobacco, grams of alcohol/week and other substances); history of depressive disorders and use of antidepressant drugs; and socio-demographic characteristics (gender, age, educational level, social class based on occupation, and marital status).

At baseline and again after 15 days, 1, 3 and 6 months, the following variables will be assessed in all participants: level of physical activity (with the patient being classified as inactive, active or partially active, using the IPAQ); self-perceived health status, using the EQ-5D questionnaire (evaluating the dimensions of mobility, self-care, usual activities, pain/discomfort and anxiety/depression, as well as a visual analogue scale of self-reported health, scored from 0 to 100); anthropometric measurements (weight, height, body mass index and waist circumference); blood pressure using an automatic digital blood pressure arm monitor; and presence of adverse events.

The following variables will be obtained in the intervention group: participants’ attendance at physical-exercise programme sessions and possible reasons for withdrawal; degree of satisfaction and acceptance of the educational programme (scale of 1 to 5 points, ranging from “very dissatisfied” to “very satisfied”); and compliance with exercise recommendations using a simple tool based on self-recording of daily physical activity (this record will be used to assess the frequency, intensity, duration and type of exercise performed).

In patients who receive treatment with antidepressant drugs, the following will be assessed: type of antidepressant (N06A Group Anatomical Therapeutic Classification); changes in treatment across the follow-up period; adherence (Morisky-Green questionnaire); and treatment satisfaction (Satisfaction with Antidepressant Treatment Questionnaire - *Cuestionario de Evaluación de la Satisfacción con el Antidepressant treatment/ESTA*).

The cost-effectiveness analysis will take into account the direct costs of both the exercise and antidepressant-treatment programmes, including staff expenses, medication, follow-up visits and monitoring.

### Statistical analysis

All statistical analyses will be performed on a blinded basis so as to ensure that the respective subjects’ group affiliations remain unknown to the assessor. After the preliminary stages of debugging, exploratory analysis, and variable categorisation or transformation have been completed, variables of interest, stratification of variables and potential confounding at baseline in the two groups will be compared, and the homogeneity of the study variables’ baseline values will be checked. An intention-to-treat analysis will be used to calculate the following parameters with their corresponding confidence intervals: absolute increase in benefit; relative increase in benefit; and number needed to treat.

The trend in the parameters of interest in the two groups will be described and compared (comparison of proportions and means in independent groups). An analysis by subgroup will be performed according to different variables, including sex, intensity of physical activity, adherence to recommendations, etc.

Finally, the effect of the intervention in terms of reducing depressive symptoms will be estimated using a logistic regression model, with statistical adjustment between possible confounding and interaction terms. The model will be interpreted on the basis of the statistical significance of the coefficients and the value of the odds ratios of the explanatory variables.

## Discussion

The results could be useful when it comes to finding a better and more effective approach to a health problem that is prevalent among older adults, and reducing the related disease burden on society as a whole. In terms of the number of patients and duration of follow-up, the proposed clinical trial is a project which easily surpasses the few studies on this subject that have been previously conducted on the elderly. Its aim is to provide scientific evidence on a therapeutic resource -physical exercise- which has undeniable health benefits and can be applied to certain health problems, such as depressive disorders, which are of great magnitude and considerable socio-economic relevance, and have a significant impact on the quality of life of older adults.

The project will make it possible to establish the type of specific physical exercise recommendations that are capable of reducing depressive symptoms among older people who meet clinical criteria for depressive disorders. Furthermore, the duration, frequency and intensity needed to ensure an efficacy similar to that of available drug treatments will be determined, thereby remedying some of the shortcomings present in the studies undertaken to date.

Should the hypothesis prove to be true, engaging in physical exercise could be regarded as a novel therapeutic resource in primary care for treating depression in older adults, and be incorporated into clinical practice guidelines along with the currently recommended pharmacological or psychotherapeutic treatments. This new therapeutic resource could have a favourable impact on the health and wellbeing of older adults, and serve to reduce health-care costs.

Among the obstacles to achieving the designated goals, mention should be made of the difficulty of conducting a trial with a high number of patients of advanced age, the need for adequate co-ordination among the different professionals, and the necessary adherence to the physical exercise programmes by the participants in order to obtain the expected benefits. The estimated sample size has been increased by 20% to offset the foreseeable loss of subjects across follow-up. Furthermore this is an open trial, seeing as what is being tested here is not a pharmacological intervention but a six-month-long physical activity programme, something that renders the use of masking techniques inviable. Even so, it is envisaged that all statistical analyses will be performed on a blinded basis, to ensure that the respective subjects’ group affiliations remain unknown to the assessor and thereby reduce any likelihood of the assessment of the effects of the intervention being biased.

In sum, it will enable the approach taken to depressive disorders in clinical practice to be changed, by offering a therapeutic alternative to existing treatments, unencumbered by the numerous adverse effects of antidepressant therapy and capable of reducing the frequent polymedication of older adults. In addition, the benefits of exercise can be extended to the cardiovascular area and musculoskeletal disorders, thus contributing decisively to more active ageing and a better overall level of health.

## Abbreviations

EQ-5D: EuroQol 5 Dimensions
ESTA: Questionnaire for the Evaluation of Antidepressant Treatment Satisfaction
GDS: Geriatric Depression Scale
HR: Heart rate
ICD: International Classification of Diseases
ICPC: International Classification of Primary Care
IPAQ: International Physical Activity Questionnaire
MADRS: Montgomery-Asberg Depression Rating Scale

## References

[1] Blair SN (2009). Physical inactivity: the biggest public health problem of the 21^st^ century. Br J Sports Med.

[2] Lim SS, Vos T, Flaxman AD, Danaei G, Shibuya K, Adair-Rohani H (2012). A comparative risk assessment of burden of disease and injury attributable to 67 risk factors and risk factor clusters in 21 regions, 1990-2010: a systematic analysis for the global burden of disease study 2010. Lancet.

[3] Gremeaux V, Gayda M, Lepers R, Sosner P, Juneau M, Nigam A (2012). Exercise and longevity. Maturitas.

[4] Moussavi S, Chatterji S, Verdes E, Tandon A, Patel V, Ustun B (2007). Depression, chronic diseases, and decrements in health: results from the world health surveys. Lancet.

[5] Blumenthal JA, Smith PJ, Hoffman BM (2012). Is exercise a viable treatment for depression?. ACSMs Health Fit J.

[6] García Herrera JM, Nogueras EV, Muñof F, Morales JM (2011). Guía de Práctica Clínica para el tratamiento de la depresión en Atención Primaria.

[7] Duclos M, Gouarne C, Bonnemaison D (2003). Acute and chronic effects of exercise on tissue sensitivity to glucocorticoids. J Appl Physiol.

[8] Cotman CW, Berchtold NC (2002). Exercise: a behavioural intervention to enhance brain health and plasticity. Trends Neurosci.

[9] Ernst C, Olson AK, Pinel JPJ, Lam RW, Christie BR (2006). Antidepressant effects of exercise: evidence for an adult neurogenesis hypothesis?. J Psychiatry Neurosci.

[10] Schuch FB, de Almeida Fleck MP (2013). Is exercise an efficacious treatment for depression? A Comment upon Recent Negative Findings. Front Psychiatry.

[11] Danielsson L, Noras AM, Waern M, Carlsson J (2013). Exercise in the treatment of major depression: a systematic review grading the quality of evidence. Physiother Theory Pract.

[12] Greer TL, Trivedi MH (2009). Exercise in the treatment of depression. Curr Psychiatry Rep.

[13] National Institute for Clinical Excellence, NICE (2010). Depression: management of depression in primary and secondary care - NICE guidance.

[14] Morey MC. Physical activity and exercise in older adults. In: UpToDate, Post TW (Ed), UpToDate, Waltham, (Consulted 11 April 2015).

[15] Institute for Clinical Systems Improvement, ICSI (2010). Health Care Guideline: Major Depression in Adults in Primary Care.

[16] Sjosten N, Kivela SL (2006). The effects of physical exercise on depressive symptoms among the aged: a systematic review. Int J Geriatr Psychiatry.

[17] Cooney GM, Dwan K, Greig CA, Lawlor DA, Rimer J, Waugh FR (2013). Exercise for depression. Cochrane Database Syst Rev.

[18] Blumenthal JA, Babyak MA, Moore KA, Craighead WE, Herman S, Khatri P (1999). Effects of exercise training on older patients with major depression. Arch Inter Med.

[19] Blumenthal JA, Babyak MA, Doraiswamy PM, Watkins L, Hoffman BM, Barbour KA (2007). Exercise and pharmacotherapy in the treatment of major depressive disorder. Psychosom Med.

[20] Blumenthal J, Sherwood A, Babyak M, Watkins L, Smith PJ, Hoffman B (2012). Exercise and pharmacological treatment of depressive symptoms in patients with coronary heart disease. J Am Coll Cardiol.

[21] Brenes GA, Williamson JD, Messier SP, Rejeski WJ, Pahor M, Ip E (2007). Treatment of minor depression in older adults: a pilot study comparing sertraline and exercise. Aging Ment Health.

[22] Mead GE, Morley W, Campbell P, Greig CA, McMurdo M, Lawlor DA (2009). Exercise for depression. Cochrane Database Syst Rev.

[23] Dinas PC, Koutedakis Y, Flouris AD (2011). Effects of exercise and physical activity on depression. Ir J Med Sci.

[24] Blake H (2012). Physical activity and exercise in the treatment of depression. Front Psychiatry.

